# Regulation of Endothelial and Vascular Functions by Carbon Monoxide *via* Crosstalk With Nitric Oxide

**DOI:** 10.3389/fcvm.2021.649630

**Published:** 2021-04-12

**Authors:** Yoon Kyung Choi, Young-Myeong Kim

**Affiliations:** ^1^Department of Bioscience and Biotechnology, Konkuk University, Seoul, South Korea; ^2^Department of Molecular and Cellular Biochemistry, School of Medicine, Kangwon National University, Chuncheon, South Korea

**Keywords:** carbon monoxide, nitric oxide, endothelial cell, heme oxygenase, nitric oxide synthase

## Abstract

Carbon monoxide (CO), generated by heme oxygenase (HO), has been considered a signaling molecule in both the cardiovascular and central nervous systems. The biological function of the HO/CO axis is mostly related to other gaseous molecules, including nitric oxide (NO), which is synthesized by nitric oxide synthase (NOS). Healthy blood vessels are essential for the maintenance of tissue homeostasis and whole-body metabolism; however, decreased or impaired vascular function is a high-risk factor of cardiovascular and neuronal diseases. Accumulating evidence supports that the interplay between CO and NO plays a crucial role in vascular homeostasis and regeneration by improving endothelial function. Moreover, endothelial cells communicate with neighboring cells, such as, smooth muscle cells, immune cells, pericytes, and astrocytes in the periphery and neuronal vascular systems. Endogenous CO could mediate the cell-cell communication and improve the physiological functions of the cardiovascular and neurovascular systems *via* crosstalk with NO. Thus, a forward, positive feedback circuit between HO/CO and NOS/NO pathways can maintain cardiovascular and neurovascular homeostasis and prevent various human diseases. We discussed the crucial role of CO-NO crosstalk in the cardiovascular and neurovascular systems.

## Introduction

The peripheral cardiovascular system coordinately communicates with the central nervous system (CNS). Neurotransmitters released from CNS regulate cardiovascular functions, and the peripheral bloodstream can affect central nervous vascular functions after CNS injury. Endothelial cells (ECs) that form a single-cell-thick layer, the endothelium, communicate with various types of cells, such as, smooth muscle cells (SMCs), immune cells, astrocytes, pericytes, neural stem cells, neurons, oligodendrocytes, Schwann cells, and microglia (1, 2). This coordinated cellular network prevents abnormal vascular permeability, blood flow, and inflammatory response to maintain vascular homeostasis and the blood–nerve barrier (3).

CO is a freely diffusible gaseous molecule that is endogenously synthesized by two heme oxygenase (HO) enzymes, HO-1 and HO-2. HO-1 and HO-2 require O_2_ and nicotinamide-adenine dinucleotide phosphate (NADPH) to catabolize heme to CO, biliverdin IX-α, and free Fe^2+^. Biliverdin is then converted into bilirubin (BR) by biliverdin reductase. HO metabolites (i.e., CO, biliverdin, Fe^2+^, and BR) possess protective roles in ECs and vascular system by displaying angiogenic capacity, mitochondria biogenic activity, and regenerative potential in ischemia/reperfusion injury models (4–7). CO produced in the vascular and nervous systems may also facilitate the crosstalk between the peripheral nervous system and the CNS through maintenance of the blood–nerve barrier and suppression of inflammatory responses.

Notably, CO can upregulate HO-1 expression and further elevates CO production in ECs and their neighboring astrocytes, resulting in a positive feedback loop between CO and HO-1 (8, 9). Thus, induction of HO-1 by several transcription factors, including nuclear factor erythroid-2-related factor 2 (NRF2), biliverdin reductase, cAMP-response element-binding protein, activator protein 1 (AP-1), and activating transcription factor 2 (ATF2), is strongly related to CO-mediated protection of ECs and neuronal cells against stress-induced injury and apoptosis (10–12).

It has been demonstrated that CO controls nitric oxide (NO) production *via* positive and negative regulation of nitric oxide synthase (NOS) in ECs and immune cells. Similar to NO, CO rapidly interacts with heme proteins such as, NOS and soluble guanylate cyclase (sGC) (13). In this review, the most important concept is that of the effects of CO on endothelial and vascular function through CO-NO crosstalk and its signaling function between endothelial cell and other surrounding cell (i.e., SMCs, pericytes, astrocytes, and macrophages) communications. The vascular protective role of CO has been demonstrated in the cardiovascular and neurovascular systems through the facilitation of angiogenesis, mitochondria function, vasorelaxation, blood–nerve barrier maintenance, anti-inflammation, and stem cell differentiation in pathophysiological conditions. Therefore, we discussed the potential role of the CO-NO axis in the regulation of cardiovascular and neurovascular functions.

## Pathophysiological Significance of CO in Endothelial Cells

The vascular system plays a critical role in maintaining systemic tissue homeostasis through a supply of O_2_ and nutrients. Exogenous or HO-dependent CO can modulate the vascular tone by directly activating sGC and regulating NOS expression and activity. We have discussed the pathophysiological effects of CO on ischemia/reperfusion and blood–nerve barrier function in terms of crosstalk with NO.

### Effects of CO on NOS

NOSs are a family of heme-containing enzymes that catalyze the production of NO from L-arginine and NAD(P)H. There are three isoforms of NOS in mammals, of which two are constitutive and one is an inducible type. Endothelial NOS (eNOS) and neuronal NOS (nNOS) are low-output Ca^2+^-dependent enzymes, whereas, inducible NOS (iNOS) is a high-output Ca^2+^-independent enzyme, which is transcriptionally induced in macrophages and hepatocytes by various inflammatory stimulants (14). However, NOSs synthesize not only NO but also superoxide ($O_{2}^{-}$) from NAD(P)H in the absence of L-arginine. NO rapidly reacts with $O_{2}^{-}$ to produce peroxynitrite (ONOO^−^), a highly reactive nitrogen species (RNS) that causes detrimental effects to the surrounding cells by directly causing cell injury and decreasing intracellular redox potential *via* oxidation of glutathione (15). Considering the role of NO in vascular function, an elevated $O_{2}^{-}$ level decreases the bioavailability of NO in ECs, leading to impairment of eNOS/NO-dependent vascular functions, including vasorelaxation and angiogenesis. CO has a dual effect on NOS activation depending on its biological concentration. Notably, physiological levels of CO increase steady-state levels of NO in ECs without affecting eNOS activity, owing to a CO-displaceable intracellular NO storage pool (16, 17). Similar to eNOS-derived NO, HO-derived CO functions as a vasodilator by directly activating sGC. However, pathological levels of CO inhibit the catalytic activity of purified eNOS by biding to its prosthetic heme group *in vitro* (18). As described previously, toxic CO concentrations in the neurovascular systems contribute to neurological, and cardiac injuries driven by NO and reactive oxygen species (ROS)/RNS (19). Because both HO-1 and NOS enzymes require NAD(P)H as a reducing equivalent, high induction of HO-1 may decrease endothelial NO production by competing with eNOS for NAD(P)H in ECs (20). However, NO also augments the cytoprotective effects of CO (21). Indeed, NO and its RNS by-products have been shown to be potent inducers of HO-1 expression and elevate CO production in a mechanism depending on the *de novo* synthesis of RNA and proteins (22), resulting in CO-mediated cytoprotective effects. These results indicate that the NOS/NO and HO-1/CO pathways coordinately improve the cytoprotective effect and endothelial function in the vasculature.

#### CO-eNOS Crosstalk

In ECs, eNOS can be activated by Ca^2+^-dependent dimerization and Akt-dependent phosphorylation, leading to improvement of enhanced NO-dependent vascular function (23). Treatment of bovine aortic ECs with the CO-releasing molecule (CORM)-2 (25 μM) stimulates eNOS phosphorylation on Ser1179 residue and eNOS dimerization, without altering eNOS expression, leading to the enhanced NO production by intracellular Ca^2+^ release through inositol triphosphate (IP3) receptor, and phosphoinositide 3-kinase (PI3K)-Akt signaling (23). Under inflammatory conditions, eNOS expression can be downregulated by inflammatory condition, although this enzyme is constitutively expressed (24). Pretreatment of human umbilical vein endothelial cells (HUVECs) with CORM-2 (200 μM) rescues tumor necrosis factor-α (TNF-α)-mediated downregulation of eNOS by inhibiting nuclear factor-kappa B (NF-κB)-responsive biogenesis of miR-31-5p and miRNA-155-5p, which target 3′-untranslational regions of eNOS transcript (24). Notably, the potent HO-1 inducer heme- and CORM-2-mediated recovery of TNF-α-induced eNOS downregulation are blocked by the HO inhibitor SnPP (24), suggesting that the HO-1/CO circuit plays an important role in the protection of ECs from TNF-α-induced inflammatory responses. Conversely, high levels of CO can act as a negative regulator of eNOS activity by interacting with its catalytic heme moiety in ECs, leading to the suppression of NO production and the elevation of vasoconstriction (17, 25). In an *in vivo* model, rats that inhaled 1,000 ppm CO in a chamber after myocardial ischemia-reperfusion induced by coronary artery occlusion for 30 min revealed phosphorylation peaks of Akt and eNOS at 4 and 12 h after inhaling CO, respectively (26). Under the same experimental condition, a myocardial cGMP level was also found due to the activation of the Akt-eNOS pathway (26). Taken together, the HO/CO axis may regulate the eNOS/NO pathway by multiple mechanisms, such as eNOS expression and post-translational regulation.

#### CO-iNOS Crosstalk

The HO-1/CO pathway is linked to the inhibition of iNOS and oxidative stress in various types of cells including ECs. Interestingly, intravital microscopy studies have revealed that iNOS-deficient mice have greater leucocyte-endothelial cell interactions than wild type mice during lipopolysaccharide (LPS)-induced endotoxemia, without altering the expression level of adhesion molecules (27), thus, indicating that the iNOS/NO axis participates in the regulation of inflammation-mediated endothelial cell function. CORM-2 treatment decreased iNOS protein levels but not cyclooxygenase-2 through peroxisome proliferator-activated receptor γ activation in LPS-treated mouse macrophage RAW 264.7 cells (28). Unlike CO, HO-1 can stabilize LPS-induced iNOS expression by preventing its lysosomal degradation in RAW 264.7 cells wherein this effect is independent of the HO metabolites such as, CO, bilirubin, and Fe^2+^ (29). In an *in vivo* model, pretreatment of rats with CO gas significantly reduced LPS-induced mortality, and CO suppresses LPS-induced iNOS and NO production in the lung and alveolar macrophages and enhances LPS-induced iNOS protein levels in the liver and hepatocytes (30). This suggests that CO differentially modulates iNOS expression in the lung and liver and that NO elicits a protective effect in the liver and production in the liver but a detrimental effect in the lung. Additionally, exposure of hepatocytes to exogenous CO resulted in rapid induction of iNOS expression *via* NF-κB activation and a subsequent increase in NO production, which are responsible for the protective effect of HO-1/CO signaling (31). In a model of myocardial ischemia/reperfusion of rat pre-exposed to urban CO pollution, CO increased iNOS expression and consequently increased NO levels, without altering expression and phosphorylation of eNOS (32). Taken together, although many *in vitro* and *in vivo* data demonstrate the protective role of CO in oxidative stress and inflammatory states, its effect may appear differentially *via* negative and positive regulation of iNOS expression in an organ-specific manner.

#### CO-nNOS Crosstalk

nNOS has been identified within atherosclerotic plaques, suggesting its role in endothelial inflammation (33, 34). nNOS has also been found to be expressed in rat vascular SMCs, and its activation upon stimulation by angiotensin II was demonstrated in spontaneously hypertensive rats; however, this effect was not observed in normotensive rats (35). These results indicate that nNOS may be involved in the regulation of vascular function and inflammation under normal and diseased conditions. Although, there is no direct evidence that CO regulates nNOS expression and activation in the vascular system, limited data have been shown in neuronal cells. The nNOS-NO system is not functional in HO-2-deficient mice and requires that CO itself be adequate to restore physiologic effects of NO. CO may enhance nNOS catalytic activity or facilitate NO release from enteric neurons (36). Direct treatment of rat embryonic neural stem cells (NSCs) with CORM-3 during the recovery phase after hypoxia/reoxygenation (H/R) showed no effect on NSC differentiation into mature neurons; instead, treatment of H/R-induced NSCs with conditioned medium from CORM-3-exposed pericytes under oxygen-glucose deprivation-induced NSC differentiation into mature neurons (6). In this study, the phosphorylated nNOS level was increased and coincided with markers for mature neurons, such as, growth-associated protein 43 (GAP43), neuronal nuclear antigen (NeuN), and neurofilament (6). These effects are abolished by NOS inhibitor, L-N^G^-Nitro arginine methyl ester (L-NAME) (6), suggesting that CO-NO crosstalk can be facilitated by cell-cell interaction at the recovery phase after ischemic injury. Consistent with these *in vitro* data, CORM-3 (4 mg/kg) injection 1 h after traumatic brain injury induces p-nNOS expression in neuronal cells including NSCs, probably through the improvement of pericyte-NSC communication (6). L-NAME administration just before traumatic brain injury abolished CORM-3-mediated neurogenesis and CORM-3-mediated improvement of motor functions (6). These results suggest that CO stimulates NSC differentiation and neurogenesis by increasing phosphorylated nNOS and also provide the possibility that CO contributes to the anti-inflammatory effect in the pathogenesis of atherosclerosis, in part *via* upregulation of nNOS expression and activity.

### Biological Function of CO in the Vasculature

**Figure 1:**
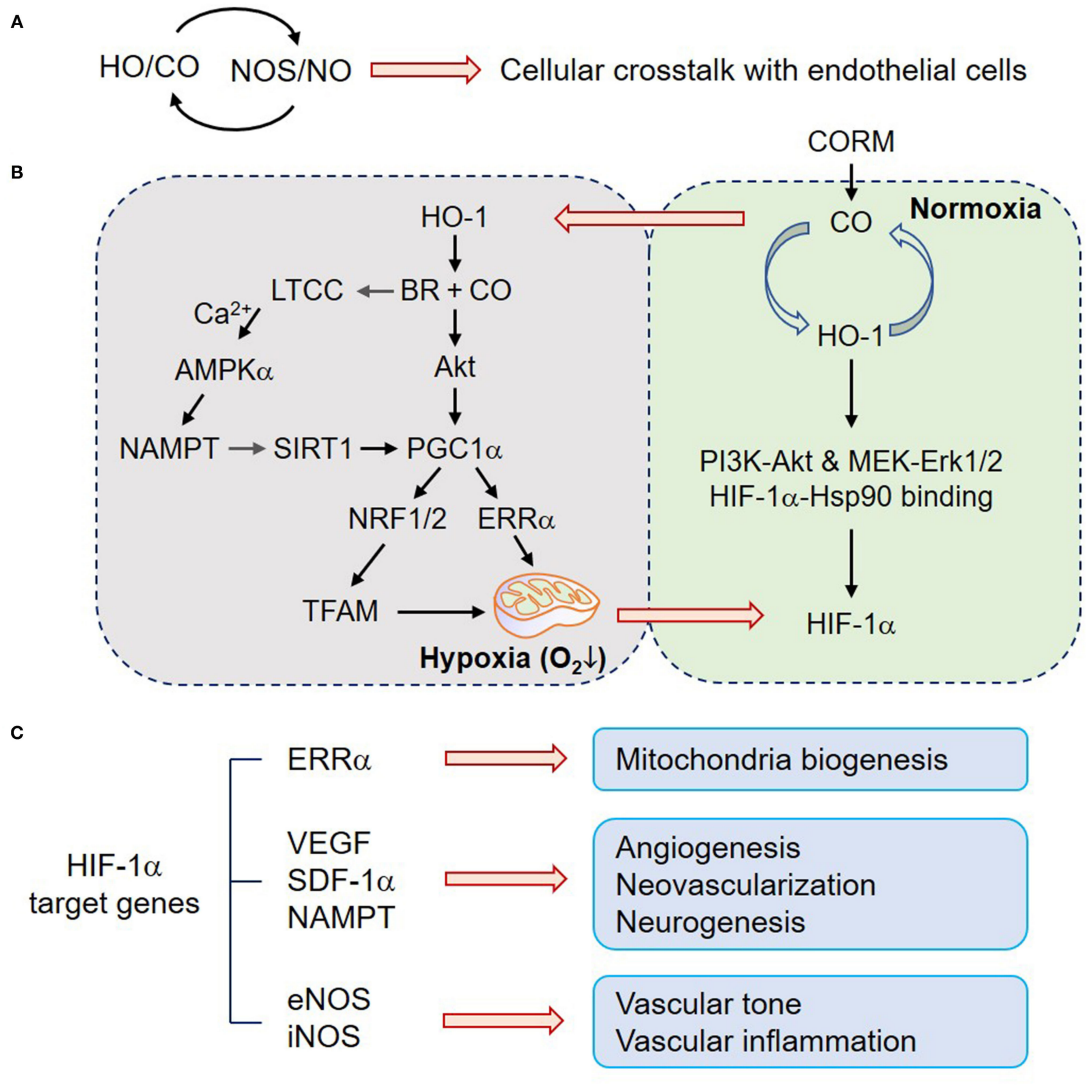
CO upregulates mitochondria biogenesis, angiogenesis, and neurogenesis. **(A)** HO/CO–NOS/NO crosstalk may play beneficial roles in ECs through cellular communications. **(B)** In normoxic astrocytes, the HO-1/CO pathway upregulates HIF-1α protein level by translational facilitation through PI3K-Akt and MEK-Erk1/2 and protein stability through HIF-1α-Hsp90 binding. In contrast, HO-1-mediated BR and CO production can induce LTCC activation, and consequent Ca^2+^ influx triggers the AMPKα-NAMPT–SIRT1–PGC-1α signaling cascade. CO also increases Akt-dependent PGC-1α activation, NRF1/2 expression, and TFAM upregulation. PGC-1α-ERRα-mediated mitochondrial biogenic activation transiently induces hypoxia (enhanced O_2_ consumption) and HIF-1α stabilization. **(C)** HO-1/CO pathway-mediated HIF-1α stabilization transcriptionally expresses its putative target genes, such as ERRα, VEGF, SDF-1a, NAMPT, eNOS, and iNOS, which are related to mitochondria biogenesis, angiogenesis, neovascularization, neurogenesis, vascular tone, and inflammation. CO, carbon monoxide; HO-1, heme oxygenase-1; BR, bilirubin; PI3K, phosphoinositide 3-kinase; MEK, mitogen-activated protein kinase kinase; Erk, extracellular signal-regulated kinase; Hsp90, heat shock protein 90; HIF-1α, hypoxia-inducible factor-1α; LTCC, L-type Ca^2+^ channel; AMPKα, AMP-activated protein kinase α; SIRT1, sirtuin 1; PGC-1α, peroxisome proliferator-activated receptor-gamma coactivator-1α; NRF, nuclear factor erythroid-2-related factor; TFAM, mitochondrial transcription factor A; ERRα, estrogen-related receptor α; VEGF, vascular endothelial growth factor; SDF-1α, stromal derived factor-1α; NAMPT, nicotinamide phosphoribosyltransferase; iNOS, inducible nitric oxide synthase.

In a neurovascular unit, ECs are surrounded by various cell types. Cell-cell communication between them may facilitate cellular metabolism and neurovascular function under physiological and pathological conditions partly through HO/CO–NOS/NO crosstalk (Figure 1A). In this section, we focused on the effects of CO on mitochondria biogenesis, angiogenesis, and blood–nerve barrier functions in pathophysiologic conditions.

#### Mitochondria Biogenesis

Mitochondria have a central role in energy metabolism and also modulate cell death by mitochondrial membrane permeabilization. CO transiently blocks cytochrome c oxidase (complex IV), leading to increased production of ROS, which subsequently act as second messengers for CO signaling. Thus, the CO-mitochondrion-ROS axis triggers activation of the redox-sensitive transcription factor NRF2 and upregulates various antioxidant enzymes, such as, glutamate-cysteine ligase and HO-1 expression, leading to an increase in cellular redox potential and CO production (37, 38). In general, nuclear NRF2 induces transcriptional gene expression by binding to the antioxidant response element (ARE) in the promoter of genes coding for antioxidant enzymes and some transcription factors. The transcription factor NRF1 associated with mitochondrial biogenesis has four AREs in its promoter, and its expression is regulated by CO-mediated NRF2 activation (37). Indeed, exogenous CO increases cardiac mitochondrial biogenesis by sequential pathways, such as, a transient increase in mitochondrial ROS production, NRF-2 activation, and NRF1 expression (37). In general, mitochondria biogenesis entails replication of mitochondrial DNA transcription by mitochondrial transcription factor A (TFAM) that is upregulated *via* peroxisome proliferator-activated receptor-gamma coactivator (PGC)-1α-dependent NRF1 synthesis (39–41). Treatment with CO-generating molecule dichloromethane increased Akt-dependent PGC-1α activation, NRF1/2 expression, and TFAM upregulation, resulting in mitochondrial biogenesis, a transient increase in intracellular O_2_ consumption (transient hypoxia), and subsequent stabilization of HIF-1α (5, 8, 42, 43) (Figure 1B). These results postulate that HO-derived CO may improve cellular metabolism *via* mitochondrial biogenesis in some types of cells, such as, cardiomyocytes and astrocytes, although, there is no direct evidence at present for the effect of CO on mitochondrial biogenesis in ECs.

#### Angiogenesis

The prominent role of CO in the vascular system is to regulate vascular relaxation, although its activity can be complex and dynamic depending on the concentrations of O_2_, NO, and other gaseous molecules. Another major biological function of CO has been considered in vessel growth and angiogenesis through O_2_-dependent (although transient) and O_2_-independent pathways, which are regulated by HIF-1α-dependent vascular endothelial growth factor (VEGF) gene expression (5, 8, 42). In addition to the role of HIF-1α in VEGF expression, estrogen-related receptor α (ERRα) also directly upregulates VEGF expression in a HIF-1α-independent manner by binding to the VEGF gene promoter (44). Therefore, it has been suggested that VEGF expression and angiogenesis can be regulated by CO in a HIF-1α-dependent or independent multiple manner. CO induces HO-1 expression in various cell types (8, 45) forming a positive feedback loop between HO-1 and CO. Indeed, HO-1/CO activates the AMPKα-SIRT1-PGC-1α-ERRα axis and consequently induces VEGF expression and secretion from astrocytes located in neighboring ECs (5), suggesting that CO improves neurovascular function *via* angiogenesis. Alternatively, the PGC-1α-ERRα axis also activates HIF-1α protein stability, leading to VEGF expression and secretion (42). This effect is likely to be highly correlated with the possibility that HO-1/CO triggers PGC-1α/ERRα-dependent mitochondria biogenesis, leading to enhanced O_2_ consumption and consequent O_2_-dependent VEGF expression (Figure 1B). Furthermore, heme metabolites, such as, CO and BR activate the L-type Ca^2+^ channel, and consequent extracellular Ca^2+^ influx activates AMPKα, leading to upregulation of nicotinamide phosphoribosyltransferase (NAMPT) expression in astrocytes (5). NAMPT-mediated enhanced NAD^+^ levels can activate NAD^+^-dependent histone deacetylase, SIRT1, which contributes to PGC-1α/ERRα-dependent, but HIF-1α-independent, VEGF expression (5). CO alone may not activate L-type Ca^2+^ channel; however, the combination of HO metabolites (i.e., CO and BR) can potentially induce L-type Ca^2+^ channel-mediated Ca^2+^ influx, consequently leading to angiogenesis *via* the AMPKα-NAMPT-SIRT1-PGC-1α/ERRα-VEGF axis (5). A chromatin immunoprecipitation assay revealed that CO preconditioning potentiates the binding capacity of HIF-1α to its putative binding region within the *ERR*α promoter in astrocytes (42) (Figure 1C). In contrast, it has been shown that CO may limit the activity of the L-type Ca^2+^ channel during hypoxia, suggesting cardioprotective effects of HO-1 can arise from an inhibitory action of CO on L-type Ca^2+^ channels (46). The reason for such a discrepancy is still unclear, although, it could be due to the differential experimental conditions, such as cell type or CO treatment (i.e., direct or preconditioning).

Exposure of renal tubular epithelial cells to low-dose chemical NO and iNOS-derived NO, similar to CO, induces the accumulation of HIF-1α under normal O_2_ conditions (47); however, a high dose of NO donor cannot display the same result in the epithelial cells (47) and abrogates hypoxia-induced HIF-1α accumulation in hepatoma and neuronal cells (48). HIF-1α is crucially involved in transcription not only of the major angiogenic growth factor VEGF but also of several other genes including eNOS (49) and iNOS (50) (Figure 1C). Considering that the HO-1/CO axis and eNOS/NO or iNOS/NO pathway are mutually regulated (24, 31, 33), the angiogenic activity of the HO-1/CO axis is likely related to HIF-1α-dependent VEGF expression in coordination with the NO pathway.

However, adenoviral overexpression of HO-1 or CO donor treatment revealed attenuated cardiac dysfunction *via* neovascularization in a murine model of myocardial infarction by recruiting or homing circulating endothelial progenitor cells (EPCs) to ischemic area, whose effect was highly correlated with VEGF expression and stromal-derived factor 1 (SDF-1) (51, 52). Collectively, these results suggest that HO-1-derived CO stimulates angiogenesis by inducing angiogenic factors and the recruitment of circulating EPCs (Figure 1C).

#### Blood–Nerve Barrier

CO plays a significant role in maintaining the junction barrier between adjacent ECs and alleviating blood-brain barrier (BBB) dysfunction following ischemic stroke, leading to maintenance of healthy vascular function in pathophysiological conditions (6, 53). Blood–nerve barrier, an endothelial barrier constructed by an extensive network of ECs, astrocytes, and neurons to form functional “neurovascular units” (3, 54), is essential for the regulation of the CNS microenvironment. Several studies demonstrated that CO reduced cerebrovascular permeability in mouse model of traumatic brain injury and malaria infection by maintaining vascular tight junction and blood–nerve barrier including BBB (6, 55). The suppressive effect of CO on cerebrovascular permeability in a mouse models of traumatic brain injury is thought to the improvement of crosstalk between ECs and other types of cells including pericytes (6). ONOO^−^ and other RNS cause vascular permeability through post-translational modification of junction-associated proteins, such as, *S*-nitrosylation of β-catenin and tyrosine-nitration of RhoGAP (56, 57). HO-1-derived CO may in part maintain vascular permeability *via* reduction of ONOO^−^ and other RNS production by inhibiting the catalytic activity of the heme-dependent ROS-generating enzyme NADPH oxidase (Nox) activity (58). Therefore, HO-1-derived CO plays a crucial role in the maintenance of vascular integrity and blood–nerve barrier function through crosstalk with the NO pathway.

## Pathophysiological Significance of CO on Vascular Function

Healthy vasculature can be regulated by surrounding cells, such as, SMCs, pericytes, and astrocytes (1, 59, 60). Additionally, the vascular functions are also affected by secreted cytokines from macrophages and microglial cells under inflammatory conditions (61). Cell-cell interactions trigger vessel protective signaling, such as, amelioration of inflammation when cells are exposed to subtoxic CO or transient HO-1 induction. Therefore, we discussed the role of CO in endothelial cell-neighboring cell interactions, specifically emphasizing vascular functions.

### EC-SMC Crosstalk

**Figure 2:**
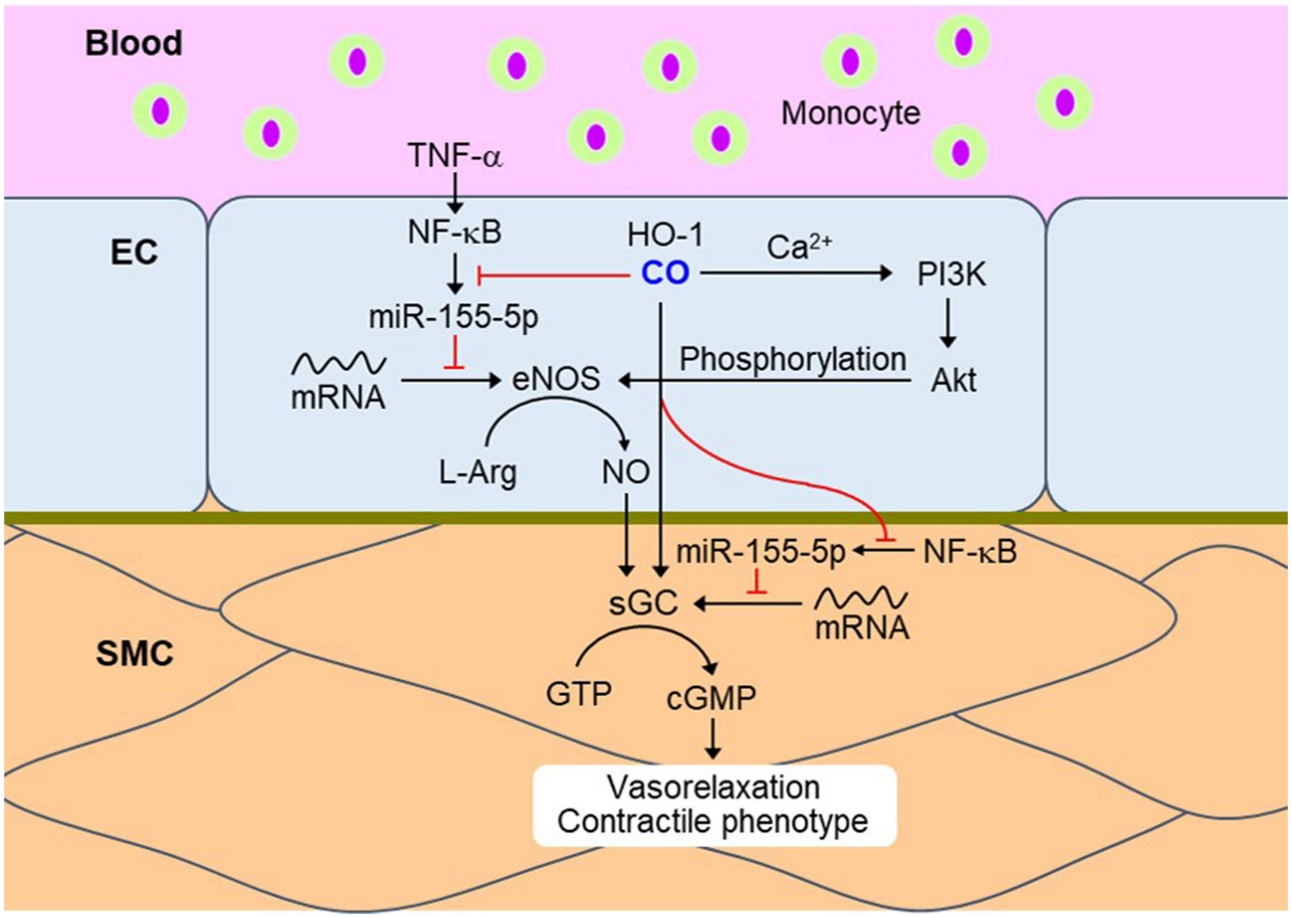
CO momentarily downregulates NF-κB signaling in ECs and SMCs during the inflammatory condition. Transient HO-1/CO pathway blocks TNF-α-mediated NF-κB activation and subsequent miR-155-5p biogenesis. miR-155-5p downregulates eNOS and sGC by targeting their transcripts, resulting in vasoconstriction. Therefore, inhibition of NF-κB-responsive miR-155-5p biogenesis by CO may recover vasorelaxation and contractile phenotype. Red dash: blockade signal by CO. NF-κB, nuclear factor-κB; EC, endothelial cell; SMC, smooth muscle cell; TNF-α, tumor necrosis factor-α; eNOS, endothelial nitric oxide synthase; L-Arg, L-arginine; sGC, soluble guanylyl cyclase; GTP, guanosine triphosphate; cGMP, cyclic guanosine monophosphate.

SMCs regulate vascular tone by driving the contraction/relaxation of the vascular wall and thus controls the diameter of the blood vessel lumen (62). Several reports show that the vasodilatory properties of HO-1-derived CO are clearly linked to the NOS-sGC-cGMP axis and the activation of Ca^2+^-dependent and voltage-dependent big conductance K^+^ channel (BK channel) in SMCs (63, 64). CO directly modulates the function of various heme proteins, such as, sGC and BK_Ca_, in which the reduced Fe^2+^ center interacts with CO (65). Binding of CO with the BK_Ca_ channel stimulates the channel activity (65). The activation of BK_Ca_ channels leads to membrane hyperpolarization, which in turn inhibits voltage-gated Ca^2+^ channels, causing SMC relaxation and vasodilation (66). Additionally, CO indirectly supports the vasodilative role through EC-SMC crosstalk. Transient HO-1/CO-mediated NF-κB inactivation is connected to vascular tone *via* upregulation of the eNOS/NO axis in ECs and subsequently increases sGCβ1-dependent cGMP production in SMCs. NF-κB activation is responsible for miR-155-5p expression, which is a key player for eNOS downregulation in HUVECs (24) and for sGCβ1 reduction in vascular SMCs in inflammatory conditions (67), consequently leading to vascular constriction. Therefore, CO-NO crosstalk induces vascular dilation through EC-SMC communication through CO-mediated downregulation of NF-κB-responsive miR-155-5p biogenesis (Figure 2).

SMC-derived factors can affect cGMP production through the transient HO-1/CO pathway. Interestingly, conditioned medium from primary cultures of rat aortic SMCs exposed to hypoxia has been shown to stimulate cGMP production, whose effect was completely abolished by tin protoporphyrin (an inhibitor of HO), or hemoglobin (a scavenger of CO and NO) (64). Vascular SMC-specific overexpression of HO-1 exerts systemic hypertension through abolishing NO-mediated cGMP production and vasodilation (68). In obese Zucker rats, excessive CO production may lead to vascular dysfunction and hypertension partly through altered NO production (69). Vascular constriction due to CO over-production is probably associated with multiple mechanisms, such as, competitive consumption of NADPH by eNOS and HO-1, degradation of the prosthetic heme moiety for NOS, and binding of CO to the heme group (70). Therefore, sustained overexpression of HO-1 may induce vasoconstriction through altered NOS inactivation; in contrast, transient HO-1/CO pathway may activate NOS/NO-mediated vasodilation.

### EC-Pericyte Crosstalk

Cardiac pericytes may be derived from the epicardial mesothelium, and brain pericytes may be derived from the neural crest (71, 72). The origin of pericytes is heterogeneous in a tissue-dependent manner. ECs expressing platelet-derived growth factor (PDGF) recruit PDGF receptor β (PGGFRβ)-expressing pericytes for vessel maturation (73). Thus, PDGF- or PDGFRβ-deficiency causes perinatal lethality secondary to lack of mural cells and vascular instability (73, 74). Additionally, PDGFRβ-deficient mice demonstrated markedly reduced total number of Notch3^+^ mural cells and decreased differentiation from pericyte precursors to coronary artery SMCs in response to Notch3 signaling (75). Moreover, angiopoietin-1 produced by pericytes binds to its receptor Tie2 on ECs, leading to stimulation of pericyte recruitment and vessel stabilization and angiogenesis (76). Therefore, pericytes play a crucial role in vascular function and homeostasis.

Impaired pericyte recruitment to ECs results in a malfunctional blood–nerve barrier and consequent cognitive dysfunctions in the CNS (77, 78). Pericyte coverage of ECs in the brain and retina is ~80–90% and is crucial for the formation and maintenance of blood–nerve barrier (79). Various protective factors (i.e., PDGF-BB) derived from pericytes may contribute to blood–nerve barrier integrity. Pericytes also communicate with oligodendrocytes, NSCs, and ECs (6, 80–82); thus, pericyte loss may lead to blockage of interactions with vessel-associated cells and consequent communications between white matter and gray matter. Inhalation of CO in a mouse model of traumatic brain injury significantly decreased nitrosative-oxidative stress-mediated apoptotic cell death in PDGFR-β^+^ pericytes (6), suggesting that CO potentially protects pericytes from unfavorable stress environments.

The HO-1/CO pathway can be triggered by PDGF exposure in mural cells (83). Treatment of brain pericytes with CORM-3 under oxygen-glucose deprivation partly recover *in vitro* and *in vivo* pericytes cell survival (6). Similar to CO, phenyl N-tert-butylnitrone (PBN), an RNS scavenger, can also ameliorate pericytes cell apoptosis 3 days after traumatic brain injury (6). In diabetic retinopathy, NF-κB activation in pericytes is a critical mechanism for eliciting the proapoptotic program (84). One of the NF-κB-downstream target molecules, matrix metalloproteinase-9 (MMP-9), is induced in brain pericytes subjected to traumatic brain injury, which is diminished by CORM-3 or PBN injection (6), implying that CO abrogates MMP-9-mediated tight junction degradation by inhibition of the NF-κB and ROS/RNS crosstalk (85). Additionally, CO triggers the pericyte-NSC crosstalk, thus, enhancing the neurogenic capacity of the adult brain after traumatic brain injury (6). Conditioned media from CO-exposed pericytes triggers NSC differentiation into mature neurons (6). Therefore, a complex set of ligand-receptor interactions between the ECs and pericytes can be modulated by the diffusible gaseous molecules, CO, and NO.

### EC-Astrocyte Crosstalk

Adenosine diphosphate results in CO production by freshly isolated piglet cerebral microvessels and astrocytes (86), suggesting endogenous CO modulates astrocytic functions. Astrocytic endfeet also possess Ca^2+^-dependent and voltage-dependent BK channels, which control capillary constriction and dilation in a Ca^2+^ concentration-dependent manner (87). Astrocytic Ca^2+^ plays a key role in signaling transfer between astrocytes, regulation of vascular tone, and modulation of neuronal activity (88). Since the HO-1/CO pathway reveals activation of L-type Ca^2+^ channel in human astrocytes, HO metabolites (i.e., CO and BR) may activate voltage-dependent BK channel through Ca^2+^ influx in astrocytes, leading to modulation of vascular dilation and neuronal activity (88).

Astrocytic HO-1 can stabilize HIF-1α protein, which regulates ERRα and VEGF in ischemic stroke mouse models (5). In an animal model of ischemic injury, excess VEGF mediates vascular permeability; however, appropriate VEGF level exerts regenerative effects, such as angiogenesis and neurogenesis partly through HO-1 upregulation (5, 89–91). Therefore, regeneration through the astrocytic VEGF-HO-1 circuit in ischemic conditions varies depending on their expression level and period and an alternate route of modulating the VEGF signaling pathway. CO-exposed astrocytes also have neuroprotective functions through brain-derived neurotrophic factor and its receptor TrkB in purinergic signaling-mediated damage condition (92). The HO-1/CO pathway inhibits amyloid β_1−42_ (Aβ_1−42_)-mediated ONOO^−^ production and Nox-derived ROS generation in astrocytes (93). NOS inhibitor diminishes Aβ_1−42_-mediated astrocyte toxicity (93), suggesting that crosstalk between astrocytic CO and NO can influence neurovascular functions, such as, blood–nerve barrier formation, angiogenesis, and neurogenesis, by modulating growth factors and neurotrophic factors (94–96).

### EC-Monocyte/Macrophage Crosstalk

**Figure 3:**
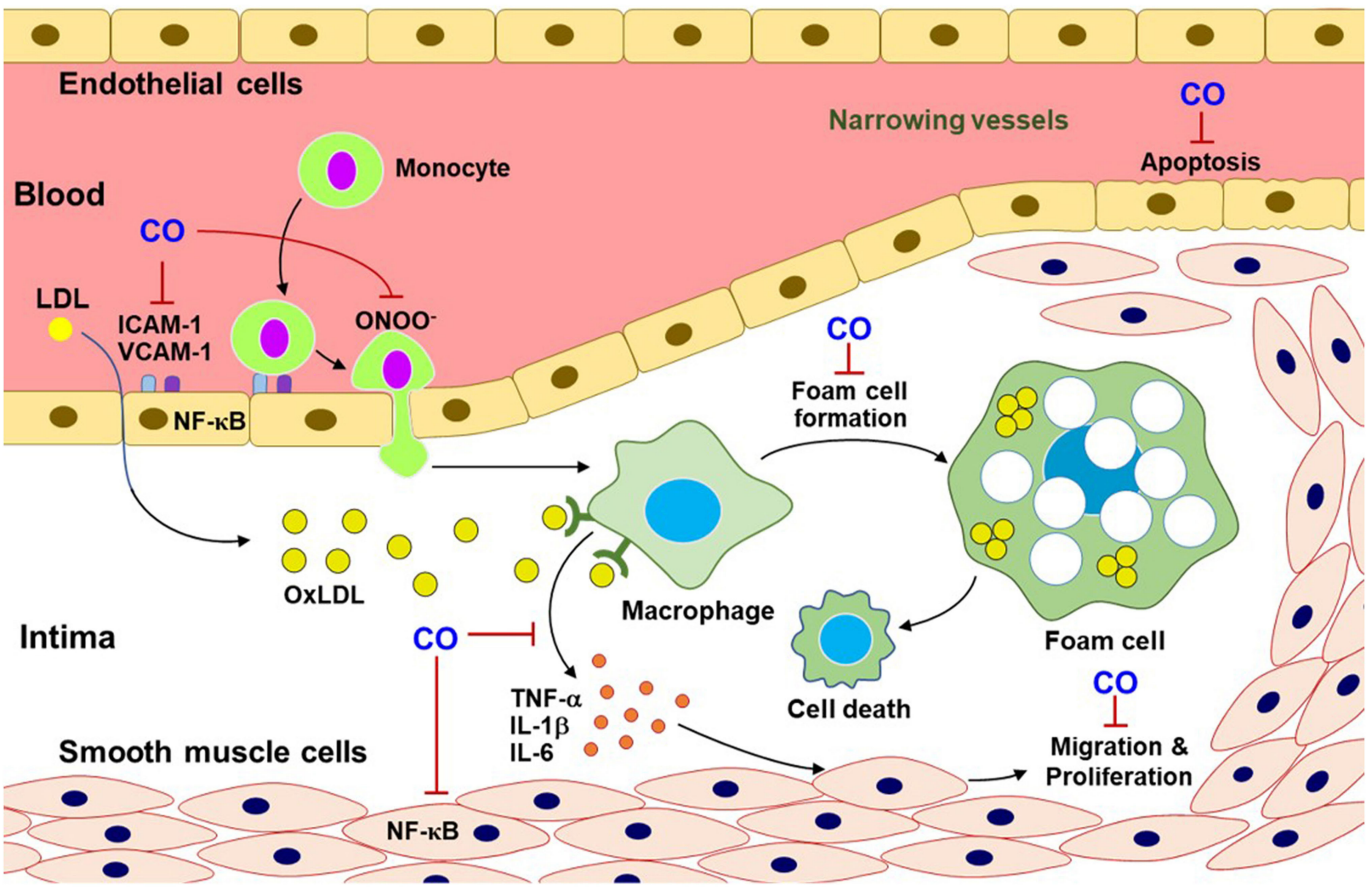
CO inhibits pro-atherosclerotic processes. CO blocks the multifaceted signaling pathways related to atherosclerotic progress. This progress can include monocyte infiltration through adhesion molecules (i.e., ICAM-1 and VCAM-1), macrophage differentiation from monocytes, foam cell formation, and inflammatory cytokine releases and ONOO^−^ production. Additionally, CO reduces EC apoptosis and SMC migration and proliferation. OxLDL, oxidized low-density lipoprotein; ICAM-1, intercellular adhesion molecule-1; VCAM-1, vascular cell adhesion molecule-1; TNF-α, tumor necrosis factor-α; IL-1β, interleukin-1β.

Recent reports display that CO or CORMs can decrease NF-κB-mediated inflammasome formation. Activated NF-κB acts as a mediator for the NOD-, leucine-rich region-, and pyrin domain-containing protein 3 (NLRP3) inflammasome in inflammatory conditions (97). LPS binding to toll-like receptor 4 in macrophage leads to the NF-κB-mediated transcriptional activation. NF-κB-dependent genes (i.e., NLRP3, pro-interleukin (IL)-1β, and pro-IL-18) then activate inflammasome assembly, leading to mature IL-1β secretion (98). Additionally, NF-κB-mediated transcription of adhesion molecules, such as, intercellular adhesion molecule-1, vascular adhesion molecule-1, and epithelial cell adhesion molecule-1, in ECs can induce monocyte infiltration into injured tissue (99, 100) (Figure 3). CORM-2 repair damages ECs through inhibiting NF-κB activation and consequently upregulating eNOS expression (24). Treatment with CO gas negatively regulates NLRP3 inflammasome activation in macrophages under LPS- and ATP-treated conditions (61). CO-mediated anti-inflammatory effects on macrophages have been demonstrated to be induced by the HIF-1α-transforming growth factor β (TGF-β) axis (101), indicating that transient increases in HIF-1α expression level by CO may play a key role in cellular adaptation for oxygen tension in inflammatory conditions. Collectively, EC-monocyte interaction can be modulated by crosstalk between CO and NO, leading to control of vascular function and inflammation.

## Role of CO in Vasculopathy

In ischemic vasculopathy, the development of agents that are effective for anti-inflammation and vascular regeneration can be used to treat vascular diseases. Healthy vasculature can protect neuronal degeneration from ischemia-mediated inflammation and cell death. Thus, we next displayed the role of CO in protective and regenerative functions in some vascular diseases, such as, cardiovascular and cerebrovascular dysfunction. The basic concept is that CO diminishes excessive NO/RNS formation and stimulates therapeutic NO production, all of which maintain and improve vascular function and homeostasis.

### CO in the Pathogenesis of Hypertension and Atherosclerosis

Pulmonary arterial hypertension (PAH) and atherosclerosis show uncontrolled SMC proliferation and a narrowing of vessel lumen (38), impeding adequate blood supply to the target tissues. Hypertension is chronic vascular disease, related to inflammatory responses, which can be reversed by CO (102). Accumulation of low-density lipoprotein in arterial walls is the main characteristic of atherosclerosis (103). The lipid-laden lesions elevated a variety of NF-κB-dependent cytokines, such as, TNF-α, IL-1β, and IL-6, through communication with other cell types (i.e., macrophages) (104). Thus, NF-κB plays a key proatherogenic factor contributing to foam cell formation, vascular inflammation, vascular SMC proliferation, arterial calcification, and plaque progression (105), which are mostly suppressed by CO (250 ppm) gas inhalation for 2 h in rat atherosclerosis model (103). Moreover, the HO-1/CO pathway suppresses EC apoptosis (104) and uncontrolled SMC migration and proliferation (106) (Figure 3). These data may support a beneficial role of the HO-1/CO pathway in repair processes in hypertension and atherosclerosis.

NO is a potent vessel protector in normal conditions; therefore, CO-NO crosstalk can inhibit the initiation of hypertension and atherosclerosis. Moderate production of NO plays a protective role in cardiac ischemia-reperfusion injury when 1,000 ppm CO is exposed to rat models, and CO-mediated protective effect was attenuated by the inhibition of p38 MAPK, PI3K, NOS, and sGC (26). In contrast, following injuries such as, ischemia and inflammation, simultaneous production of NO and excessive ROS in the immune or vascular system, leading to cytotoxic ONOO^−^ generation, can trigger the pathogenesis of hypertension and atherosclerosis. Nox2-mediated mitochondria-derived ROS production in ECs contributes to angiotensin II-induced hypertension (107, 108). ROS can induce eNOS uncoupling, resulting in decreased eNOS-derived NO synthesis and EC function, is a risk factor of the pathogenesis of hypertension (109). Surged NO and ONOO^−^ cause injury of ECs and other vascular cells (110). Maintenance of eNOS-derived NO production or decreased ROS-mediated ONOO^−^ generation by HO metabolites may protect the cardiovascular system in severe inflammatory conditions (111).

### CO in Stroke and Traumatic Brain Injury

**Figure 4:**
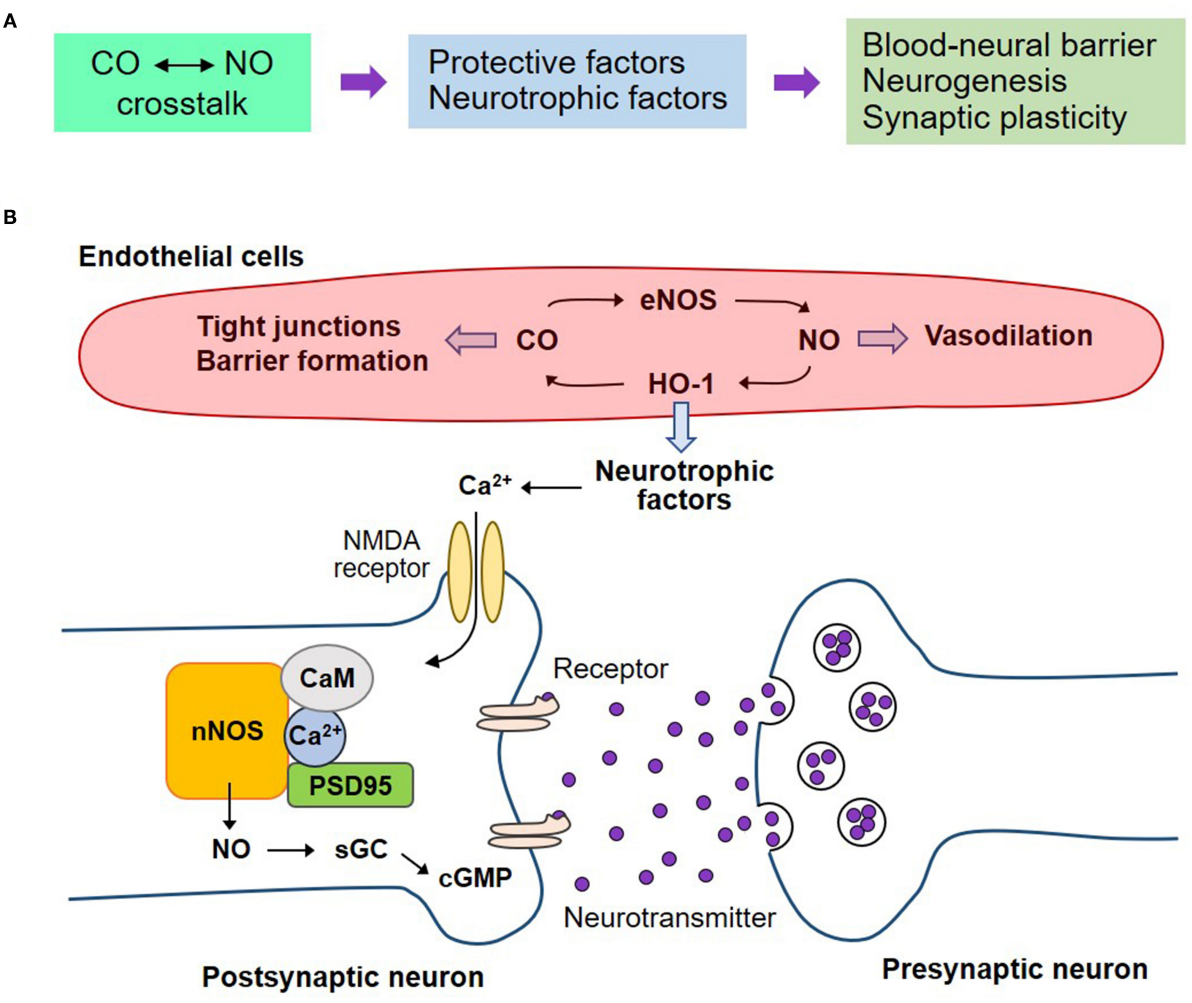
Regenerative effects of CO on the neurovascular system by crosstalk with NO. **(A)** The crosstalk between CO and NO may potentiate the secretion of neurotrophic factors, consequently leading to blood-neural barrier maintenance, neurogenesis, and synaptic plasticity on the neurovascular unit. **(B)** Healthy blood vessels are maintained by moderate levels of CO by crosstalk with eNOS/NO pathway. NO reciprocally upregulates HO-1 expression, leading to CO production. The interplay of CO with NO affects vascular function by inducing blood-neural barrier and vasodilation. The endothelial cell-mediated neurotrophic factors may induce nNOS-mediated NO production and consequent sGC activation and cGMP production. These pathways can stimulate neurogenesis and synaptic plasticity. nNOS, neuronal nitric oxide synthase; CaM, calmodulin; PSD95, post-synaptic density 95; NMDA receptor, N-methyl-D-aspartate receptor.

A subtoxic dose of CO has a dual function in the NOS/NO signaling pathway in neurodegenerative diseases, such as, stroke and traumatic brain injury (6, 53, 112). CORMs suppress ROS over-production and NOS/NO-mediated ONOO^−^ generation, resulting in anti-inflammatory responses. A CORM ALF-186 mitigates ischemia-reperfusion injury-induced sGC inactivation and NF-κB-mediated inflammatory cytokine expression, such as, TNF-α and IL-6, in the retina and serum of rats (112). CORM-3 treatment decreased infarct volume in a mouse model of ischemic stroke, also showing decreased inflammatory cytokine levels (53). Later, CO boosts the nNOS/NO signaling pathway, leading to healthy blood vessels, neurogenesis, and synaptogenesis in traumatic brain injury (6) (Figures 4A,B). In this model, L-NAME administration for NOS inhibition abolishes CORM-3-mediated neuronal differentiation, and maturation of NSCs and neurogenesis (6). nNOS is bound to N-methyl-D-aspartate receptors in the post-synaptic neurons through the adaptor protein post-synaptic density 95 (113, 114). The CO–NO crosstalk may stimulate neuronal activity and neurogenesis partly *via* a Ca^2+^/calmodulin-mediated nNOS/NO pathway (6) and formation of the sGC–cGMP axis (13). Healthy blood vessels are maintained by moderate levels of CO *via* crosstalk with eNOS/NO pathway (60). NO reciprocally upregulates HO-1 expression, leading to CO production (22, 115). In all, interplay between CO and NO potentially affects vascular function by inducing blood–nerve barrier and vasodilation (Figure 4B).

In the acute phase, endogenous or exogenous CO promotes the integrity of BBB, thus facilitating tissue repair in the pathological conditions of cerebrovascular diseases (55, 116). Maintenance of BBB can help neuronal survival and functions, such as, neurogenesis and synaptogenesis, by stimulating the secretion of neurotrophic factors from ECs and their surrounding cells (i.e., SMCs, pericytes, astrocytes, and microglia) (Figure 4B). Additionally, CO-mediated production of angiogenic factors, such as, VEGF and SDF-1, promotes vascular regeneration supplying sufficient O_2_ and nutrients (117). A low dose of CO activates the eNOS/NO axis, resulting in vascular dilation (60).

Then, CO exhibits an increase in mature neuronal markers, such as, GAP43, NeuN, and microtubule-associated protein 2, in the mouse models of stroke and traumatic brain injury (6, 53), demonstrating adult neurogenesis. This suggests that CO has a protective effect against stroke and traumatic brain injury.

## Summary and Future Perspectives

CO has multiple roles in the vascular system by itself or in physiological and pathological conditions in a NO-dependent manner. NO is a free radical molecule that interacts with various biological molecules, such as, proteins, nucleic acids, protein-containing heme, and iron-sulfur cluster groups, and thiol-containing bioactive compound, while CO is not a free radical, but interacts with ferrous heme iron. However, the biological function of CO in the vascular system overlaps in many ways with that of NO, including vasodilation, anti-inflammation, cytoprotection, and apoptotic cell death. Therefore, these CO functions are considered to be elicited by crosstalk with NO. Since CO and NO are generated by the catalytic reaction of HO-1 and eNOS in the vascular system, respectively, the positive circuit or crosstalk between the HO-1/CO and eNOS/NO pathways, the iNOS/NO, or nNOS/NO system can play a critical function in the maintenance of cardiovascular and neurovascular homeostasis and prevention of the pathogenesis of human diseases. Sometimes, CO is attributable to the pathogenesis of some diseases. This detrimental effect may be due to the high concentration of NO, prolonged exposure, and cell type-specific phenomena. Despite these problems, some clinical trials using inhaled CO gas has been conducted and showed some effectiveness (www.clinicaltrials.gov) in patients with PAH (Identifier: NCT02368171), kidney transplantation (Identifier: NCT00531856), and postoperative ileus (Identifier: NCT01050712). Another CO clinical translation test also shows protection of islet cells from death after autologous islet transplantation in patients with chronic pancreatitis when the CO-saturated medium is provided for harvesting islets (Identifier: NCT02567240) (www.clinicaltrials.gov). To extend our knowledge of CO-mediated clinical applications and biological functions, it is necessary to understand the interrelationship between CO and NO in the cardiovascular and neurovascular systems under physiological and pathological conditions.

## Author Contributions

YC and Y-MK wrote the manuscript. Y-MK received funding and supervised the project. All authors contributed to the article and approved the submitted version.

## Conflict of Interest

The authors declare that the research was conducted in the absence of any commercial or financial relationships that could be construed as a potential conflict of interest.
